# A Combined LC-MS and Immunoassay Approach to Characterize Preservative-Induced Destabilization of Human Papillomavirus Virus-like Particles Adsorbed to an Aluminum-Salt Adjuvant

**DOI:** 10.3390/vaccines12060580

**Published:** 2024-05-26

**Authors:** Ria T. Caringal, John M. Hickey, Nitya Sharma, Kaushal Jerajani, Oluwadara Bewaji, Sarah Brendle, Neil Christensen, Saurabh Batwal, Mustafa Mahedvi, Harish Rao, Vikas Dogar, Rahul Chandrasekharan, Umesh Shaligram, Sangeeta B. Joshi, David B. Volkin

**Affiliations:** 1 Department of Pharmaceutical Chemistry, Vaccine Analytics and Formulation Center, University of Kansas, 2030 Becker Drive, Lawrence, KS 66047, USA; ria.caringal@ku.edu (R.T.C.); hickey1@ku.edu (J.M.H.); nsharma@mriglobal.com (N.S.); kaushyj@gmail.com (K.J.); darraog@gmail.com (O.B.); joshi@ku.edu (S.B.J.); 2 Department of Pathology, College of Medicine, Pennsylvania State University, 500 University Drive, Hershey, PA 17033, USA; ganzelly@gmail.com (S.B.); waipu6514@gmail.com (N.C.); 3 Serum Institute of India Pvt. Ltd., Pune 411028, India; saurabh.batwal@seruminstitute.com (S.B.); mustafa.mahedvi@seruminstitute.com (M.M.); harish.rao@seruminstitute.com (H.R.); vikas.dogar@seruminstitute.com (V.D.); rahul.chandrasekharan@seruminstitute.com (R.C.); umesh.shaligram@seruminstitute.com (U.S.)

**Keywords:** vaccine, stability, adjuvant, preservatives, Alhydrogel, human papillomavirus, virus-like particles, ELISA, cysteine

## Abstract

During the multi-dose formulation development of recombinant vaccine candidates, protein antigens can be destabilized by antimicrobial preservatives (APs). The degradation mechanisms are often poorly understood since available analytical tools are limited due to low protein concentrations and the presence of adjuvants. In this work, we evaluate different analytical approaches to monitor the structural integrity of HPV16 VLPs adsorbed to Alhydrogel™ (AH) in the presence and absence of APs (i.e., destabilizing m-cresol, MC, or non-destabilizing chlorobutanol, CB) under accelerated conditions (pH 7.4, 50 °C). First, in vitro potency losses displayed only modest correlations with the results from two commonly used methods of protein analysis (SDS-PAGE, DSC). Next, results from two alternative analytical approaches provided a better understanding of physicochemical events occurring under these same conditions: (1) competitive ELISA immunoassays with a panel of mAbs against conformational and linear epitopes on HPV16 VLPs and (2) LC-MS peptide mapping to evaluate the accessibility/redox state of the 12 cysteine residues within each L1 protein comprising the HPV16 VLP (i.e., with 360 L1 proteins per VLP, there are 4320 Cys residues per VLP). These methods expand the limited analytical toolset currently available to characterize AH-adsorbed antigens and provide additional insights into the molecular mechanism(s) of AP-induced destabilization of vaccine antigens.

## 1. Introduction

Vaccine access and affordability, especially in low- and middle-income countries (LMICs), remains a challenge to combat emerging and established infectious diseases. For example, although vaccination against human papillomavirus (HPV) has successfully lowered mortality and morbidity rates from associated cervical and oropharyngeal cancers worldwide [1,2,3,4], licensed HPV vaccines are less available in LMICs, in part due to high costs and limited manufacturing capacity. Currently, HPV vaccines are only available in single-dose presentations (i.e., Gardasil^®^, Gardisil9^®^, Cervarix^®^, and Cecolin^®^). The development of multi-dose vaccines is one proven formulation strategy to facilitate lowering vaccine costs and improving global distribution [5]. Such multi-dose formats require the addition of antimicrobial preservatives (APs) to prevent microbial ingress from repeated needle withdrawals from a vaccine container used over multiple days [6].

The most widely used AP in licensed vaccines is thimerosal (TH) [7]; however, this organomercurial compound can decrease the potency of certain vaccines including inactivated polio (IPV) and HPV vaccines [8,9]. The molecular mechanism of TH-induced degradation of a vaccine antigen starts with TH decomposition in aqueous solutions into thiosalicylate and ethylmercury, with the latter capable of forming a reversible S-Hg coordinate bond with accessible cysteine residues in proteins comprising the vaccine antigen [10,11]. These adducts can cause local and global structural alterations leading to disruption of key epitopes and loss of vaccine potency [12]. For example, TH inactivates HPV vaccines resulting in substantially lower immunogenicity due to a loss of epitopes and distinct alterations to morphology of the antigen (human papillomavirus virus-like particles, HPV VLPs) [13]. Thus, alternative APs to TH are required to develop stable multi-dose formulations of HPV vaccines.

Multi-dose formulation development of aluminum-salt adjuvanted HPV VLPs with alternative APs has recently been described by our lab [14,15,16] as well as by others [17]. Our work was focused on a quadrivalent mixture of antigens (HPV6, 11, 16, and 18 VLPs) formulated with an aluminum-salt adjuvant (Alhydrogel™, AH). There are numerous analytical challenges to monitoring the stability of HPV VLPs in the presence of AH adjuvant and APs (see Discussion), and we down-selected in vitro potency (competitive ELISA assays) and antimicrobial effectiveness (modified pharmacopeia tests) to facilitate multi-dose formulation development [14]. Both empirical and statistical (DOE) screening studies with hundreds of candidate multi-dose formulations were then performed. Seven different APs found in approved parenteral products were evaluated [6,14,18], both individually and in combination, including chlorobutanol (CB), m-cresol (MC), 2-phenoxyl ethanol (2-PE), benzyl alcohol (BA), phenol, (PH) methylparaben (MP), and propylparaben (PP) [15]. Stable candidate HPV VLP multi-dose formulations included certain APs (CB, 2-PE, BA, or 2-PE + BA), while other APs (MC, PH, and parabens) displayed suboptimal stability [16].

Interestingly, this rank-ordering of the storage stability profiles of HPV VLPs vs. AP type did not correlate with global destabilization effects measured by DSC [14,16] or with reports from other groups evaluating various HPV VLPs from different sources [13,17]. Thus, while these previously described analytical methods were successful in enabling the development of multi-dose formulations for specific HPV antigens, they were insufficient to elucidate the physicochemical mechanism(s) of degradation. The latter are needed to better understand these observed instability trends across different APs and HPV antigens. Therefore, new analytical approaches are needed to better characterize structural changes within vaccine antigens adsorbed to an aluminum adjuvant, especially when induced by APs (other than TH) that have been reported to interact with proteins via non-specific and non-covalent mechanisms [19,20,21].

In this work, we characterized the structural integrity and stability of AH-adsorbed HPV16 VLPs formulated either in the presence or absence of two APs, one non-destabilizing (chlorobutanol, CB) and the other destabilizing (m-cresol, MC). We focused on the HPV16 L1 serotype since we had access to an extensive number of conformational and linear mAbs previously reported to bind known epitopes within the HPV16 VLPs [22,23]. First, we performed extensive immunoassay evaluations of AP-induced destabilization of AH-adjuvanted HPV16 VLPs using a series of mAbs against specific epitopes on HPV16 VLPs. Second, we developed an LC-MS peptide mapping assay, combined with a differential alkylation strategy, to monitor the accessibility/redox state of the Cys residues located in each L1 protein within an HPV16 VLP. The results are discussed in the context of formulation development of AH-adsorbed recombinant protein antigens, including (1) expanding the limited analytical toolset currently available for their characterization and (2) providing better structural insights into the mechanism(s) of their AP-induced destabilization.

## 2. Materials and Methods

Materials used in this work are described in the Supplemental Methods. Briefly, HPV16 VLPs in solution were produced by the Serum Institute of India, Pvt. Ltd. and stored frozen at −80 °C until use. The conformational mAbs (H16.V5, H263.A2, H16.7E, and H16.E70) and linear mAbs for HPV16 (H16.J4, H16.S1, H16.H5, H16.B20, and H16.D9) utilized in the competitive ELISA assays were produced by the laboratory of Professor Neil Christensen (Penn State University), as described previously [22,23]. Descriptions of formulation workflows are provided in the Supplemental Methods including HPV VLP antigen adsorption to aluminum-salt adjuvant (Alhydrogel™, AH) and the setup of accelerated stability studies in formulation buffers at different pH values. Finally, descriptions of the analytical methods performed to evaluate the AH-adsorbed HPV VLPs are provided in the Supplemental Methods (and in some cases are also described previously, [14,15,16]) including differential scanning calorimetry (DSC), competitive ELISAs, SDS-PAGE, cysteine accessibility assay (i.e., differential alkylation, enzymatic digestion, and LC-MS peptide mapping), and data visualization tools.

## 3. Results

### 3.1. Stability Profile of AH-Adsorbed HPV16 VLPs at Elevated Temperatures between pH 6.5 and 7.4

Previous studies in our laboratories with AH-adsorbed quadrivalent HPV VLPs in the presence of one or more antimicrobial preservatives (APs), in a formulation buffer at pH 6.5, demonstrated instability under accelerated storage conditions (40 °C, 3 m and 25 °C, 12 m) as measured by in vitro potency assays (competitive ELISAs; see below). In this work, as an initial set of experiments, we further evaluated the inherent stability profile of each AH-adsorbed HPV VLP in a quadrivalent formulation as a function of pH and temperature in the same formulation buffer (20 mM histidine, 250 mM NaCl, 0.025% polysorbate 80) in the absence of APs. The goal was to first identify baseline conditions to generate stress samples (i.e., pH, temperature, HPV VLP serotype) for the subsequent development of analytical methods for mechanistic studies of AP-induced degradation. By using the same competitive ELISA assays described previously in our multi-dose formulation work [14,15,16] (i.e., binding of one HPV-type-specific antibody for each of the HPV L1 serotypes 6, 11, 16, and 18; see Supplemental Methods), we observed that the AH-adsorbed HPV16 VLP was the most susceptible L1 serotype to pH and temperature effects. For example, after stressed storage for up to 5 weeks (50 °C, pH 6.5–7.4), the binding to the HPV16-specific antibody was lost after ~2 weeks, while the values for the HPV6, HPV11, or HPV18 VLPs remained >80% for binding to their respective HPV-type-specific mAbs (Figure S1). Based on these results in the absence of APs, we down-selected AH-adsorbed HPV16 VLPs for this work.

**Figure 1:**
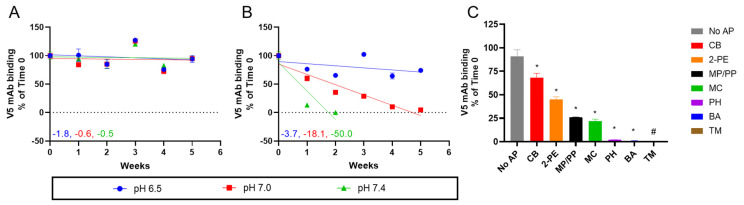
Stability profile of AH-adsorbed HPV16 VLPs as a function of temperature, pH, and AP as determined by a competitive ELISA assay measuring V5 mAb binding. The AH-adsorbed HPV16 VLPs were prepared in formulation buffer at pH 6.5 (blue), 7.0 (red), or 7.4 (green), and stability was monitored over 5 weeks when stored at (**A**) 4 °C or (**B**) 50 °C in the absence of APs. The error bars represent the range from two replicates, and the slopes of linear regression fits (percent binding loss per week relative to Time 0) are reported. The dotted lines represent no V5 mAb binding. (**C**) Stability of AH-adsorbed HPV16 L1 VLPs in presence of various APs (27 mM CB; 72 mM 2-PE; 11 mM MP + 1 mM PP; 28 mM MC; 53 mM PH; 93 mM BA; or 0.25 mM TH) was measured after 24 h at pH 7.4 and 50 °C (see panel legend for indicated AP). The error bars represent 1 SD from three replicates, and the number symbol (#) denotes that no V5 binding was measured. An asterisk symbol (*) denotes that the V5 binding in the AP containing sample was significantly (*p* < 0.05) lower compared to the control (no-AP) sample. Descriptions of formulation composition and stability study conditions are provided in the Supplemental section.

The stability profile of the AH-adsorbed HPV16 VLPs was then evaluated in more detail at different temperatures and pH values and in the presence and absence of various APs. During 4 °C storage for up to 5 weeks, no notable differences in antibody binding (neutralizing conformational mAb V5) were observed across the pH range of 6.5–7.4 (Figure 1A). Incubation at 50 °C, however, resulted in dramatic differences in the antibody binding of HPV16 VLPs between pH 6.5 and 7.4 (Figure 1B). The relative loss in antibody binding was ~4, ~18, or ~50% per week, at pH 6.5, 7.0, or 7.4, respectively. Given the prominent decrease in the inherent stability of AH-adsorbed HPV16 VLPs formulated at pH 7.4 and incubated at 50 °C for 24 h, these stress conditions were applied for all subsequent studies.

AH-adsorbed HPV16 VLPs were then incubated under these stress conditions with individual APs, and the loss of V5 antibody binding activity was measured. In the no-AP control sample, V5 binding decreased slightly (~9% lower) compared to Time 0 (T0) (Figure 1C). The AH-adsorbed HPV16 VLPs were least destabilized by CB with ~68% of relative antibody binding remaining, followed by 2-PE (~45%), MP/PP (~26%), and MC (~22%). Little to no (≤2%) V5 binding was measured in the HPV16 samples containing PH, BA, or TH. The reduction in V5 binding to AH-adsorbed HPV16 VLPs was statistically significant (*p* < 0.05) in the presence of each tested AP compared to the control (no-AP) sample. Based on these results, two APs (CB and MC) were down-selected for further study, which displayed relatively good and poor stability profiles, respectively, for AH-adsorbed HPV16 VLPs under these stress conditions. Interestingly, the addition of CB and MC resulted in relatively good and poor stability profiles, respectively, during our previously reported real-time and accelerated stability studies with AH-adsorbed quadrivalent HPV VLPs in the same formulation buffer at pH 6.5. (i.e., storage at 4 °C and 25 °C for up to 2 and 1 years, respectively) [16].

### 3.2. Stability Profiles of AH-Adsorbed HPV16 VLPs in Presence of CB or MC and Correlations with DSC and SDS-PAGE Results

**Figure 2:**
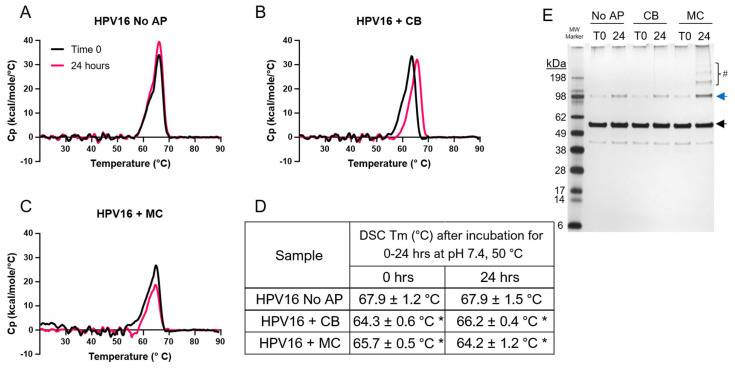
DSC and SDS-PAGE analysis of AH-adsorbed HPV16 VLP samples, before and after stressed storage and with and without AP addition. Representative DSC thermograms of AH-adsorbed HPV16 VLPs (**A**) in the absence of an AP at Time 0 (black trace) and after incubation for 24 h at 50 °C (pink trace) or in the presence of (**B**) 27 mM CB or (**C**) 28 mM MC. (**D**) Summary of the average thermal melting temperature values of AH-adsorbed HPV16 VLPs with the error representing 1 SD from six replicates. An asterisk symbol (*) denotes that the measured Tm values in the HPV16 + CB (or MC) samples were statistically (*p* < 0.05) lower compared to the control (0 h, HPV16 no-AP) sample. (**E**) Representative SDS-PAGE analysis of AH-HPV16 VLP samples under reducing conditions at Time 0 (T0) or after 24 h at 50 °C (24). The L1 monomer (black arrow) or dimer (blue arrow) band and higher-molecular-weight bands (number symbol, #) are indicated. The relative abundance of the dimer band in the MC containing sample after thermal stress (24 h, 50 °C) was ~20%, while the corresponding higher-molecular-weight bands were below the LOQ. Descriptions of formulation buffer (pH 7.4), AP concentration, and stability study conditions are provided in the Supplemental Methods section.

The overall conformational stability of AH-adsorbed HPV16 VLPs in the absence (no-AP) or presence of APs (CB or MC) was determined by differential scanning calorimetry (DSC) analysis in formulation buffer, pH 7.4. In the absence of APs, a single endothermic transition was observed both in an unstressed sample (at T0) and the same sample after 24 h of incubation at 50 °C and pH 7.4 (Figure 2A) with a thermal melting temperature (Tm) value of ~68 °C. For the time zero samples (T0) of AH-adsorbed HPV16 VLPs in the presence of APs, DSC analysis revealed small but statistically significant (*p* < 0.05) destabilization effects with Tm values of ~64 and ~66 °C (i.e., ΔTm values of ~4 and ~2 °C) in the presence of CB and MC, respectively. This result did not correlate with the trends in the storage stability data (i.e., MC was much more destabilizing than CB). In contrast, DSC analysis of the stressed samples (24 h, 50 °C, pH 7.4) showed that MC addition resulted in a smaller total area of the thermogram (i.e., the apparent enthalpy value) and a larger ΔTm value (~4 °C) compared to the CB and no-AP samples (~2 °C) (Figure 2B–D). These DSC data with stressed samples of AH-adsorbed HPV16 VLPs are consistent with their storage stability profiles (MC vs. no-AP and CB samples).

The same AH-adsorbed HPV16 VLP stress samples were also analyzed by reduced SDS-PAGE (Figure 2E). The migration of the prominent band in HPV16 samples (no-AP, CB, or MC), at both T0 or after 24 h at 50 °C, was between the 62 and 49 kDa molecular weight markers and consistent with the theoretical size of the HPV16 L1 monomer (~55 kDa) [14]. Faint higher-molecular-weight bands were visually observed in five of six samples but could not be quantified since the levels were below the LOQ. In one sample (24 h 50 °C MC sample; far right lane in gel), the level of the presumed L1 covalently linked dimer band (migrating near the ~98 kDa molecular weight marker) was quantitated at ~20%. No notable accumulation of lower-molecular-weight species was observed in the three samples before or after stress treatment, indicating that AP-induced destabilization was not due to fragmentation of the HPV16 L1 protein.

Taken together, the DSC and SDS-PAGE results with stressed samples of AH-adsorbed HPV16 VLP samples (no-AP, CB, or MC) qualitatively displayed trends consistent with their respective stability profiles (competitive ELISA). These trends, however, were modest in their extent and were difficult to interpret since their levels approached experimental uncertainty. Although these results suggest that physicochemical alterations to the AH-adsorbed HPV16 VLPs occur under these stressed conditions, better analytical tools are needed to elucidate specific degradation pathways.

### 3.3. Stability of AH-Adsorbed HPV16 VLPs in the Presence of CB or MC Measured by Immunoassays with a Panel of HPV16-Specific mAbs toward Conformational and Linear Epitopes

**Figure 3:**
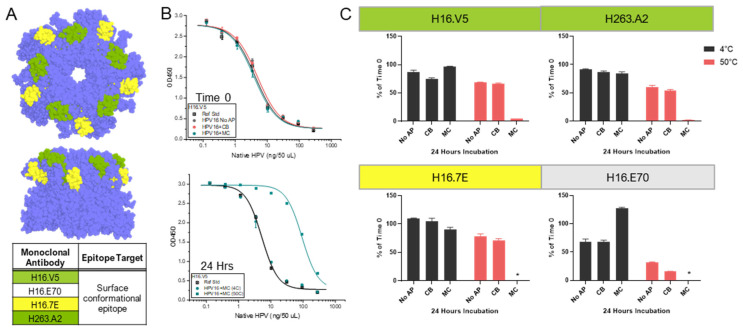
Binding of specific mAbs (V5, A2, 7E, E70) to conformational epitopes on HPV16 VLP antigen formulated with AH adjuvant (adsorbed) in the absence or presence of APs. (**A**) Illustrative model of the known conformational mAb epitope binding sites on the HPV16 L1 pentamer (colored blue). The model was generated in *PyMol* Ver 2.0.3 using HPV16 L1 pentamer (PDB ID 3J7G) and known binding epitopes from the literature [22,23]. Please note that the binding epitopes for H16.V5 and H263.A2 are similar and are thus color-coded the same. The binding epitope for H16.E70 (no color) is currently unknown. (**B**) Representative competitive ELISA binding curves of AH-adsorbed HPV16 VLP samples with the H16.V5 mAb at Time 0 (**top**) or after 24 h at 4 °C or 50 °C (**bottom**) in the absence (no-AP) or presence of 27 mM CB or 28 mM MC. (**C**) Summary of antibody binding results of each individual conformational mAb to AH-adsorbed HPV16 VLPs after 24 h at 50 °C relative to Time 0 in the absence (no-AP) or presence of 27 mM CB or 28 mM MC. Descriptions of formulation buffer (pH 7.4) and stability study conditions are provided in the Supplemental Methods section. The error bars represent the range from two replicates, and the asterisk (*) denotes that no mAb binding was measured.

**Figure 4:**
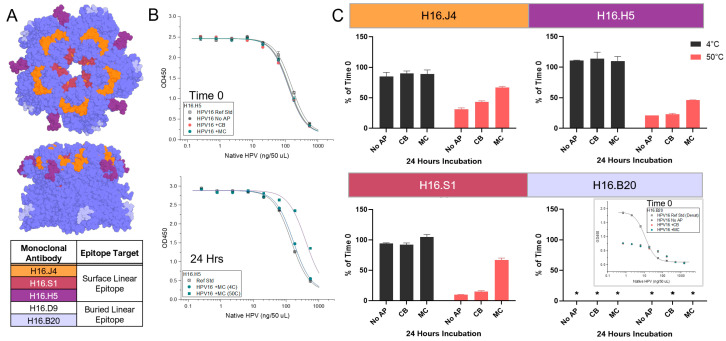
Binding of specific mAbs (J4, H5, S1, B20) to linear epitopes on HPV16 VLP antigen formulated with AH adjuvant (adsorbed) in the absence or presence of APs. (**A**) Illustrative model of the known linear mAb epitope binding sites on the HPV16 L1 pentamer (colored blue). The model was generated in *PyMol* Ver. 2.0.3 using HPV16 L1 pentamer (PDB ID 3J7G) and known binding epitopes from the literature [22,23]. The binding epitope for H16.D9 (no color) is currently unknown. (**B**) Representative competitive ELISA binding curves of AH-adsorbed HPV16 L1 VLP samples to H16.H5 mAb at Time 0 (**top**) or after 24 h at 4 °C or 50 °C (**bottom**) in the absence (no-AP) or presence of 27 mM CB or 28 mM MC. (**C**) Summary of the antibody binding results of each individual linear mAb to AH-adsorbed HPV16 L1 VLPs after 24 h at 50 °C relative to Time 0 in the absence (no-AP) or presence of 27 mM CB or 28 mM MC. Inset for B20: representative binding curves for Time 0. Note that the extensively denatured HPV16 VLP sample (positive control—inset) displayed a sigmoidal curve, while all the other samples, including the reference sample, did not bind to the B20 mAb. There was no binding observed for the D9 mAb as well (Figure S2). Descriptions of formulation buffer (pH 7.4) and stability study conditions are provided in the Supplemental Methods section. The error represents a range from two replicates, and the asterisk (*) denotes that no mAb binding was measured.

To further investigate the nature of these potential physicochemical changes (see above) induced by CB and MC with the AH-adsorbed HPV16 VLPs at the regional structural level, an expansive panel of HPV16-specific mAbs, many with known epitopes, was employed. The mAbs that bind conformational epitopes on HPV16 VLPs included V5, A2, 7E, and E70. The mAbs that bind linear epitopes on the surface of HPV16 VLPs consisted of J4, H5, and S1, while the mAbs that bind the buried linear epitopes were B20 and D9. A summary of experimental conditions performed to optimize the competitive ELISA assays using each of these mAbs is provided in Supplementary Materials Table S1. The location of the binding epitopes on HPV16 L1 pentamers (72 L1 pentamers form an HPV VLP) for most of these mAbs (except E70 and D9) have been identified previously and are displayed schematically in Figure 3A (for mAbs binding conformational epitopes) and Figure 4A (for mAbs binding linear epitopes) [22,23].

The AH-adsorbed HPV16 VLPs were incubated under stress conditions (24 h at 50 °C in formulation buffer at pH 7.4) in the absence (no-AP) or presence of CB or MC. The antibody binding levels with each of the mAbs described above were measured and compared to T0 values and to samples stored at 4 °C. First, samples were tested with the V5, A2, or 7E mAbs against conformational epitopes (Figure 3B,C), and the trends observed in the relative amount of antibody binding in each of the three formulations (no-AP, CB, MC) were evaluated. For example, after 24 h at 4 °C, ~0–25% loss was seen compared to the T0 values. The relative binding of the fourth conformational mAb (E70, epitope location unknown) displayed trends of decreased (~30% loss) values in the no-AP or CB samples and elevated values in the MC sample (~30% increase), a result likely influenced by assay variability. For the same samples stored under stress conditions (24 h at 50 °C), trends were observed of moderate losses (~20–45%) in the no-AP and CB samples when tested using the V5, A2, or 7E mAbs. For the E70 mAb, larger (~70% or ~85%) trends in the losses of relative binding were noted for the no-AP or CB samples, respectively, under stressed conditions. For the stressed MC samples, even larger losses were observed with only negligible binding when tested with these four mAbs (≥~95% loss). These results suggest that MC causes distinct regional changes (i.e., conformational epitope loss) on the surface of the HPV16 VLPs compared to the no-AP and CB samples.

For the same AH-adsorbed HPV16 VLP stability samples, trends in the relative binding to mAbs against the surface linear epitopes (J4, H5, and S1) were also evaluated (Figure 4B,C). For the 24 h at 4 °C samples, the observed trends were overall similar (≤~15% change) to the values measured at T0. After 24 h at 50 °C, however, the relative binding of the three mAbs to the no-AP or CB samples showed trends of notable decreased binding (by ~60–90% relative to T0). Conversely, the relative binding of these same mAbs displayed trends of more modest decreased binding (~35–55%) in samples with MC. Finally, no antibody binding was measured when tested using the mAbs against buried linear epitopes (B20) (Figure 4C) or D9 (Figure S2) under any formulation conditions or timepoints for the AH-adsorbed HPV16 VLPs. In fact, the binding of these two mAbs against buried linear epitopes was only observed with extensively denatured HPV16 VLPs (see inset of Figure 4C).

In total, these combined competitive ELISA results show that upon exposure to stress conditions (pH 7.4, 50 °C for 24 h), the AH-adsorbed HPV16 VLPs display subtle alterations to local epitope sites within the VLP (i.e., small conformational changes at the epitope level within the pentamers of the HPV16 VLPs). Concomitantly, no substantial structural alterations (i.e., accessible buried linear epitopes or notable fragmentation) were observed. These structural destabilization effects are relatively more apparent in the presence of MC compared to the no-AP or CB samples, the latter which were overall similar to each other.

### 3.4. LC-MS Peptide Mapping Method to Monitor the Accessibility/Redox State of Cys Residues in the L1 Protein in AH-Adsorbed HPV16 VLP Samples

**Figure 5:**
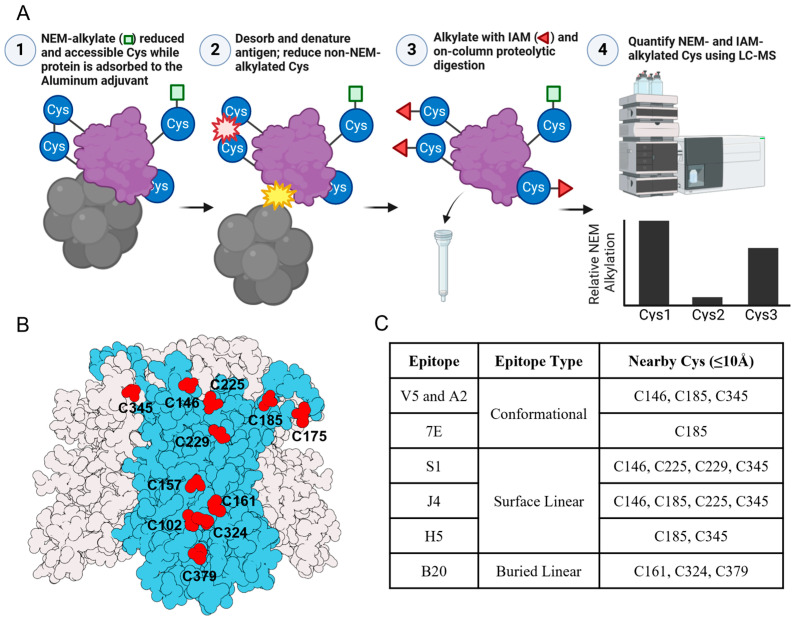
Overview of the LC-MS peptide mapping assay workflow to measure the redox state and accessibility of Cys residues in HPV VLP antigens adsorbed to an aluminum-salt adjuvant. (**A**) Schematic of the key experimental steps with images created with BioRender.com (accessed 24 May 2023). (**B**) Illustrative model of HPV16 L1 pentamer (colored grey) in which one L1 monomer is colored blue and the positions of 11 out of the 12 Cys (C428 not modeled in crystal structure) are indicated in red. The image was created using *ProteinImager* (https://3dproteinimaging.com, accessed 21 April 2023) and an HPV16 L1 pentamer structure (PDB ID 2R5H) [25]. (**C**) Summary of Cys residues within 10 Å relative to one or more residues comprising each known conformational or linear epitope binding site for indicated mAbs.

We now describe an LC-MS peptide mapping assay to monitor the accessibility and/or redox state of the Cys residues in AH-adsorbed HPV16 VLP samples (see next section for molecular description of primary structure of the L1 protein that comprises the HPV VLP). Two alkylating agents are used to differentially alkylate Cys residues in the L1 protein, and then a commercial kit is utilized to generate LC-MS-compatible peptides. The method is performed in four steps, as described schematically in Figure 5A. In Step 1, N-ethylmaleimide (NEM) is the first alkylating agent used to modify accessible and free Cys residues in the AH-adsorbed HPV16 VLP sample, followed by quenching of the reaction and multiple buffer exchanges. Please note that only free sulfhydryl groups in the Cys (-SH) residues are alkylated with NEM, while modified, non-disulfide-bonded Cys residues (e.g., sulfenic, sulfinic, or sulfonic acid) would not be alkylated [24]. In Step 2, the protein antigen is denatured and desorbed from the aluminum adjuvant, and the remaining Cys residues (e.g., those in disulfide bonds) are reduced (i.e., samples are boiled at ~100 °C in the presence of the detergent SDS and the reducing agent TCEP). In Step 3, the second alkylating agent, Iodoacetamide (IAM), is added to modify the non-NEM-alkylated Cys residues and thus flag Cys residues involved in disulfide bond formation. The sample is proteolyzed and prepared for LC-MS analysis. In Step 4, the enzymatically generated peptides (containing IAM- and/or NEM-alkylated Cys) are identified and quantified (see Supplemental Methods section).

AH-adsorbed HPV16 L1 VLPs were an ideal model to demonstrate the potential and utility of this LC-MS peptide mapping Cys accessibility assay with adjuvanted protein antigens. Each HPV VLP contains 360 L1 proteins arranged in 72 pentamers. The HPV16 L1 primary structure contains 12 Cys residues that are distributed throughout the protein’s three-dimensional structure, as shown schematically in Figure 5B [25]. Therefore, this LC-MS-based Cys accessibility assay monitors the accessibility and/or redox state of the 4320 cysteine residues within an HPV16 VLP (i.e., each VLP contains 360 L1 proteins, and each L1 protein contains 12 Cys residues). Two Cys residues (C175 and C428) are primarily involved in an inter-pentameric disulfide bond, and the remaining 10 Cys are in the reduced state (free thiols) [13,26]. Three Cys residues (C146, C185, C345) are located near the conformational epitopes, while eight Cys residues (C146, C161, C185, C225, C229, C324, C345, C379) are within 10Å (~3X the distance of a hydrogen bond) of a linear epitope (Figure 5C) [27].

### 3.5. Stability of AH-Adsorbed HPV16 VLPs in the Presence of CB or MC as Measured by LC-MS Peptide Mapping Assay for Cys Residue Accessibility

**Figure 6:**
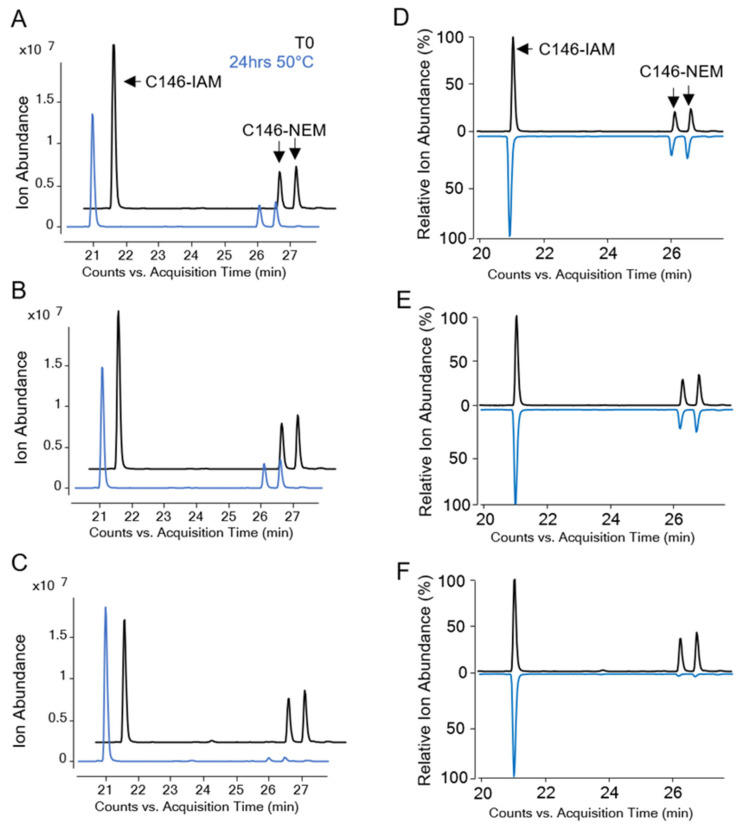
Representative extracted ion chromatograms of IAM- or NEM-alkylated C146 in the E145-K152 peptide generated from an AH-adsorbed HPV16 VLP sample at Time 0 (T0, black traces) or after incubation for 24 h at 50 °C and pH 7.4 (blue traces). Samples were incubated in the absence of an AP (panels (**A**,**D**)) or in the presence of 27 mM CB (panels (**B**,**E**)) or 28 mM MC (panels (**C**,**F**)). Panels (**A**–**C**) display the ion abundance of the IAM- or -NEM-alkylated E145-K152 peptide, while panels (**D**–**F**) display the relative percent of ion abundance. Please note that the T0 chromatograms were offset for easier visualization.

The LC-MS peptide mapping assay described above was utilized to evaluate the same unstressed (T0) and stressed (pH 7.4, 50 °C for 24 h) samples of AH-adsorbed HPV16 VLPs with and without AP (CB, MC) addition. The relative abundance of each IAM- and NEM-alkylated Cys within the HPV VLP antigen for each sample was determined. The LC-MS peptide mapping digestion method resulted in an HPV16 L1 primary sequence coverage of ≥~92%, including 100% coverage of all 12 Cys residues. Representative extracted ion chromatograms of a representative HPV16 L1 peptide (E145-K152 containing C146) are shown (Figure 6). Tandem MS/MS confirmed the identities of the single peak at ~21 min and two late-eluting peaks between 26 and 27 min as the modified E145-K152 peptide containing an IAM- or NEM-alkylated C146, respectively. NEM alkylation can result in diastereomers that elute as two distinct peaks in the RP-HPLC chromatogram, which was observed for this NEM-alkylated E145-K152 peptide [28].

**Figure 7:**
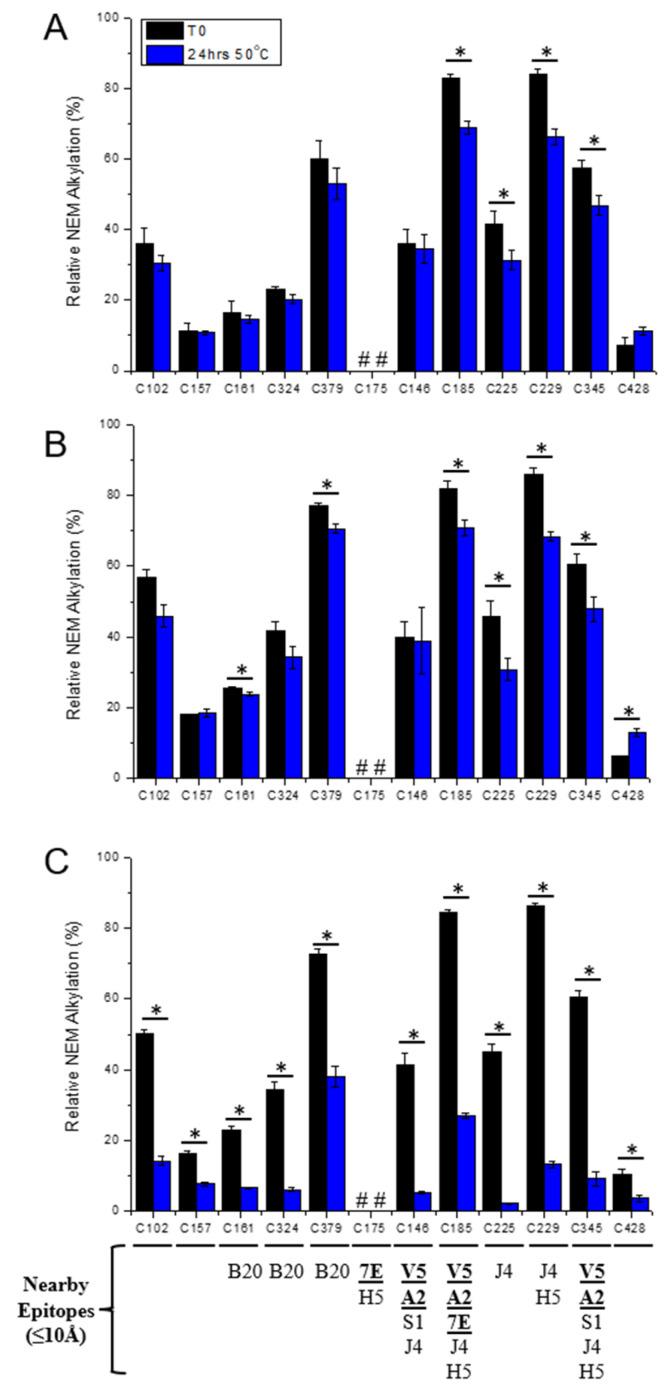
Summary of LC-MS peptide mapping quantitation of NEM-alkylated peptides containing Cys residues as generated from samples of unstressed and thermally stressed AH-adsorbed HPV16 VLP samples in the absence or presence of APs. Relative ion abundance of each NEM-alkylated Cys residue containing peptides generated from HPV16 L1 protein comprising the HPV16 VLPs is shown (**A**) in the absence of an AP or in the presence of (**B**) 27 mM CB or (**C**) 28 mM MC. Samples were evaluated both at time zero (T0) and after incubation for 24 h at 50 °C. The number (#) symbol denotes that no NEM-alkylated C175 was detected. The asterisk (*) symbol shows a significant difference (*p* < 0.05) between the relative NEM alkylation levels at T0 vs. 24 h at 50C. Error bars represent 1 SD from triplicate samples. The Cys residues found within the epitope binding site of various mAbs are shown at the bottom. Conformational mAbs are in bold and underlined; the rest are surface linear or buried linear mAbs.

Similar extracted ion chromatograms were generated for other peptides that contained each of the Cys residues found in the HPV16 L1 protein. Then, the relative abundances of IAM- and NEM-alkylated Cys residues were determined and compared (Figure 7). For example, the relative ion abundance of the IAM- and NEM-alkylated C146 peaks (described above) were plotted for each AH-adsorbed HPV16 VLP sample (see X-axis bar chart in Figure 7 for C146 residue). Similar levels were observed between the no-AP or CB samples at T0 and after thermal stress (~35–40% NEM alkylation, Figure 7A,B). In the presence of MC, however, while the relative abundance of NEM-alkylated C146 was similar at T0 (~40%), NEM alkylation decreased significantly (*p* < 0.05) in the thermally stressed sample (~5%, Figure 7C). Please note that the total ion abundance of the C146 containing peptide did not change in the stressed (24 h, 50 °C) sample containing MC. The abundance of the NEM-alkylated C146 peptide decreased, and the abundance of IAM-alkylated C146 increased concordantly (Figure 6C,F).

As summarized in Figure 7, the relative ion abundances of IAM- and NEM-alkylated Cys residues were then similarly measured for all 12 Cys residues within the L1 protein comprising the (AH-adsorbed) HPV16 VLP sample at T0 and after incubation for 24 h at 50 °C for the no-AP, CB, and MC samples. As expected, across all samples and storage conditions, ≤~10% NEM alkylation was measured for the two Cys residues known to be involved in the inter-pentameric disulfide bond (C175 and C428). For samples at T0, the relative ion abundance of each NEM-alkylated Cys was overall consistent between many of the no-AP, CB, and MC samples, except for five of the Cys residues (C102, C157, C161, C324, C379), which were ~5–20% higher in the CB sample and ~5–15% higher in the MC sample (compared to the no-AP sample). From the HPV16 L1 crystal structure (PDB ID 2R5H), these five Cys residues are located more towards the inner surface of the L1 pentamer and closer to the VLP interior rather than the exterior surface. The slightly higher NEM alkylation in these five Cys residues (C102, C157, C161, C324, and C379) suggested that the VLPs were more permeable to the NEM and/or the conformational movements on the exterior surface were more dynamic (see Discussion).

For the AH-adsorbed HPV16 VLP samples incubated for 24 h at 50 °C, either without AP or in the presence of CB (Figure 7A and Figure 7B, respectively), relative NEM alkylation levels across the 12 Cys residues in the HPV L1 protein were overall either unchanged from T0 or slightly lower (~0–20% decrease). After thermal stress, four Cys residues (C185, C225, C229, C345) were significantly (*p* < 0.05) lower without AP or in the presence of CB for 24 h at 50 °C compared to T0; concomitantly, two additional Cys residues (C161, C379) were lower, to a smaller extent but significantly (*p* < 0.05), in the presence of CB. These results indicate that at elevated temperatures, in the presence or absence of CB, only a marginal impact on the accessibility of the Cys residues throughout the HPV16 L1 protein was observed. In contrast, in the presence of MC for 24 h at 50 °C, relative NEM alkylation levels decreased significantly (*p* < 0.05) and, to a larger extent, in most of the 12 Cys within HPV16 and especially in Cys near (≤10 Å) one or more residues comprising a known epitope (Figure 7C). For example, NEM alkylation in C146, C185, or C345 that are located near the neutralizing V5 epitope decreased by ~35%, ~60%, or ~50%, respectively. The significant (*p* < 0.05) decrease in NEM alkylation for most of the HPV16 L1 Cys residues following a 24 h at 50 °C incubation with MC indicated that this AP lowered the accessibility of the Cys residues, likely through structural alterations on each HPV pentamer surface.

## 4. Discussion

### 4.1. Analytical Challenges Previously Encountered during Multi-Dose Formulation Development of AH-Adsorbed HPV VLPs

Formulation development of a multi-dose HPV vaccine candidate is technically challenging due to the requirements of an aluminum-salt adjuvant (improves immunogenicity) and APs (mitigate microbial growth) that together limit the number of analytical tools available. For example, conventional spectroscopic-based methods are ineffective due to the substantial light scattered by aluminum-salt agglomerates (1–20 µm) as well as interferences from aromatic components found in most APs used in approved parenteral products [6,29]. Analysis of HPV VLP antigens is also technically difficult due to their multivalent nature (e.g., between 2 and 9 different HPV L1 serotypes in commercial HPV vaccines) and low doses (~tens of µgs) [30,31,32,33]. Moreover, antigens adsorbed to aluminum-salt adjuvants tend to be more difficult to remove over time [29]. Finally, the antigen’s molecular composition is complex (e.g., each HPV VLP serotype is a ~60 nm capsid composed of 360 L1 protein monomers that are organized into 72 L1 pentamers connected through inter-pentameric disulfide bonds [34]).

Taken together, the complexity of the composition of candidate multi-dose formulations of adjuvanted, multivalent HPV VLPs can obfuscate the interpretation of results from most commonly used analytical assays. In our previous multi-dose HPV VLP formulation development work [14,15,16], we evaluated numerous analytical methods and only four were down-selected: two assays for the integrity of the antigen (competitive ELISA to measure relative antigenicity and SDS-PAGE for protein content) and two assays for the APs (an antimicrobial effectiveness assay and an RP-UHPLC for AP concentration). While these four analytical methods were successful in developing promising candidate multi-dose HPV vaccine formulations, such techniques were insufficient to understand the mechanisms related to AP-induced instability, especially for antigen instability caused by non-TH APs whose interactions with proteins are generally non-specific and non-covalent in nature [19,20,21].

In our previous HPV vaccine multi-dose formulation studies, we evaluated if DSC measurements of the overall conformational stability of AH-adsorbed HPV16 VLPs, in the presence of eight different APs found in licensed parenteral products [14], could explain the observed storage stability trends. For example, the destabilization trends evaluated by DSC showed that CB or MC addition to AH-adsorbed HPV16 VLPs each significantly lowered Tm values by 4–5 °C. During long-term stability studies, however, CB multi-dose formulations were much more stable than those containing MC as measured by the loss of key epitopes (competitive ELISA using HPV16-specific conformational mAb, see below). These results demonstrated that for AH-adsorbed HPV VLPs in the presence of different APs, monitoring of the global structural stability by DSC is not a suitable technique to rank-order or predict long-term storage stability (e.g., at 25 °C, 1 year and 4 °C, 2 years).

In this work, we accelerated AH-adsorbed HPV16 VLP degradation kinetics using stress conditions by raising the solution pH (pH 7.4 vs. previous studies at pH 6.5) and temperature (50 °C, 24 h). In contrast to previous studies, we not only performed DSC analysis with T0 samples but also after stress. Furthermore, we evaluated the results from SDS-PAGE with these stressed samples. Similar to the results described above, we observed a lack of correlation between DSC (T0 samples) and competitive ELISA (during stress storage) results when comparing samples in the presence of no-AP, CB, or MC. In contrast, when comparing DSC and SDS-PAGE results of stressed samples, a modest correlation in their stability profile by competitive ELISA was noted. The large differences in observed in vitro potency losses for these three samples of AH-adsorbed HPV16 VLPs, however, are unlikely accounted for by the relatively small structural changes seen by these two commonly used techniques (e.g., small differences in Tm values by DSC and low levels of covalently linked aggregates by SDS-PAGE). Therefore, new analytical tools are needed to better characterize physicochemical changes occurring in a multi-dose formulation of a vaccine antigen adsorbed to an aluminum-salt adjuvant.

### 4.2. Immunoassays with a Panel of mAbs That Bind Different Conformational and Linear Epitopes on HPV16 VLPs

Immunochemical assays are a common analytical tool used to monitor the potency of HPV VLP vaccine candidates [35]. For example, the extent of antigen binding to neutralizing antibodies, such as V5 for HPV16 VLPs, correlate with immunogenicity results in animal models and human clinical trials [35]. As applied to vaccine formulation development, Miao and colleagues used single serotype-specific neutralizing mAbs to screen six APs to develop a multi-dose formulation of a bivalent (HPV16 and HPV18) HPV vaccine candidate [17]. Non-neutralizing mAbs can also be used cooperatively with neutralizing mAbs to develop vaccine antigen multi-dose HPV VLP formulations. For example, Chen et al. and Huang et al. employed sizable panels of neutralizing and non-neutralizing mAbs to better understand the impact of primarily one AP (TH) with a quadrivalent or monovalent HPV vaccine candidate, respectively [8,13]. Using the established mechanism of TH-induced antigen destabilization (adduct formation between ethylmercury and reduced Cys on a protein), the immunochemical results in combination with electron microcopy images enabled the authors to infer structural changes on the HPV VLP in the presence of TH [10,11].

Unlike these previous studies that explored AP-induced HPV destabilization using an antigen alone (i.e., no adjuvant or aluminum-salt adjuvant was dissolved) in their ELISA assays, we developed competitive ELISAs to investigate the impact of two individual APs (CB and MC) directly with AH-adsorbed monovalent HPV16 VLPs. While developing a competitive ELISA with an aluminum-adsorbed antigen is inherently more complex than sandwich ELISA formats used for antigen alone, a key benefit of this technique is that it allows for direct measurements of immunochemical perturbations of the adsorbed antigen. This format is especially useful for dissolution-resistant aluminum-salt adjuvants (e.g., AH adjuvant used in this work; [36]). Furthermore, the previously established binding epitopes of the nine mAbs used in this work facilitated the use of their binding profiles to infer the regional effects directly with AH-adsorbed HPV16 VLPs [22,23]. This work, along with the studies reported above, demonstrates the advantage of using broad panels of mAbs (if available) to develop HPV VLP vaccine candidate formulations.

### 4.3. LC-MS Peptide Mapping Assay for Cys Accessibility and Redox State in Recombinant Protein Antigens

Current methodologies to generate residue-level LC-MS analysis of aluminum-salt-adsorbed vaccine antigens are time consuming and potentially have limited quantification of certain critical quality attributes [37]. For example, a traditional LC-MS peptide mapping workflow for aluminum-salt-adsorbed antigens consists of subjecting the sample to SDS-PAGE and then cutting protein bands of interest for subsequent proteolytic digestion and peptide analysis [38]. In addition to the extra time and reagent requirements, peptide recovery from in-gel digestion can be inconsistent and result in artificial PTMs (e.g., deamidation, oxidation) [39,40]. Another conventional approach uses high concentrations of salt (e.g., 0.2 M sodium phosphate to increase ionic strength and change the surface charge of aluminum-hydroxide adjuvants) and/or denaturant (e.g., 7.5 M guanidine hydrochloride to unfold the protein) to desorb the antigen (from the aluminum-salt adjuvant) for subsequent enzymatic processing and LC-MS analysis [41,42]. One complication of this methodology is that the strength of the interaction between an antigen and aluminum-salt adjuvant can increase over time [29]. Consequently, less antigen is desorbed, which could obfuscate the identification/quantification of destabilizing and/or potency-altering post-translational modifications.

In contrast, the LC-MS peptide mapping assay described in this study differs from the traditional approaches for MS analysis of aluminum-salt-adsorbed antigens (as described above) by using a combination of SDS, sodium phosphate, TCEP, and high temperature to thoroughly desorb HPV16 from the Alhydrogel™ adjuvant. The antigen was then isolated, buffer-exchanged, and digested using a straightforward commercial kit. In addition, this LC-MS peptide mapping assay is relatively quick (~3 h to generate HPV16 L1 peptides ready for LC-MS identification/quantification from the AH-adsorbed drug product) and requires minimal material (~20 µg antigen per analysis).

The LC-MS peptide mapping assay used herein to evaluate the accessibility/redox state of the 12 Cysteine residues in the HPV16 L1 protein could also be used in the future as a general characterization workflow to monitor critical quality attributes of either aluminum-salt-adsorbed protein or vaccine antigens in solution, including post-translational modifications, peptide fragmentation, and glycosylation. For example, we have previously utilized a similar LC-MS-peptide-mapping-based free thiol assay to identify and correlate an observed loss in in vitro potency (SRID assay) of an unadjuvanted recombinant HA influenza vaccine candidate during storage with the loss of reduced Cys residues (i.e., disulfide bond formation) in the cytosolic domain of a recombinant hemagglutinin antigen [43].

In the current study with an aluminum-salt-adsorbed HPV16 VLP antigen, no notable differences in the ion abundance values of the generated peptides were observed between the various samples, a result which indicated that a similar amount of antigen was desorbed from the aluminum-salt adjuvant across all conditions tested. Future work will focus on further establishing the quantitative nature of the ion abundance value readouts including implementing internal standards as well as comparisons to UV–visible detection. While beyond the scope of this study, the unique combination of the antigen desorption and peptide generation steps in this LC-MS method would likely not be impacted by increases in the strength of the interactions between an antigen and aluminum-salt adjuvant, for example, as may occur during long-term storage, and future studies are warranted to support this hypothesis.

### 4.4. Nature of the Interaction of m-Cresol and Chlorobutanol with AH-Adsorbed HPV16 VLPs

**Figure 8:**
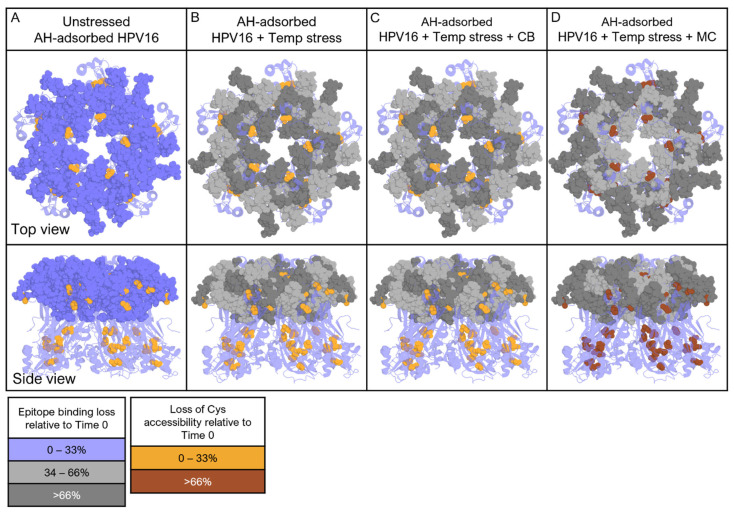
Summary schematic of HPV16 L1 pentamer antigen within AH-adsorbed HPV16 VLP samples exposed to different stress conditions as shown by (1) epitope-specific changes observed from competitive ELISA using conformational and surface linear mAbs (purple) and (2) Cys residue accessibility changes from LC-MS peptide mapping quantitation (orange). Four storage conditions are shown with AH-adsorbed HPV16 VLP samples (left to right): (**A**) unstressed (control), (**B**) elevated temperature stress (50 °C, 24 h, pH 7.4), (**C**) elevated temperature stress and CB addition, and (**D**) elevated temperature stress and MC addition. Light grey or dark grey coloring indicates a 34–66% or >66% loss of mAb binding; brown indicates a loss of Cys accessibility under the specified condition relative to Time 0. Each HPV16 L1 pentamer contains five HPV L1 proteins, and a total of 72 HPV pentamers self-assemble to form one HPV16 VLP (see text). Due to visualization limits, epitopes for buried linear mAbs are not shown.

To better understand how two non-TH APs (CB and MC) structurally destabilize HPV16 VLPs adsorbed to an aluminum adjuvant, we exposed AH-adsorbed HPV16 VLP samples to different stress conditions and then evaluated the structural integrity changes in the antigen by (1) epitope-specific changes observed from competitive ELISA using conformational and surface linear mAbs and (2) Cys residue accessibility changes from LC-MS peptide mapping quantitation. Figure 8 schematically summarizes the structural changes observed by these two analytical approaches on the exterior surface of the HPV16 pentamer (there are five L1 protein monomers per pentamer and 72 pentamers per VLP) for the unstressed AH-adsorbed HPV16 VLP sample (Figure 8A) as well as in response to stressed storage conditions (24 h at 50 °C and pH 7.4), in the absence of APs (Figure 8B), and in the presence of either CB (Figure 8C) or MC (Figure 8D). The binding epitopes for both conformational and linear mAbs are colored purple and the Cys residues are colored orange for easier visual interpretation. We use the results for the HPV16 pentamer in the unstressed sample of the AH-adsorbed HPV16 VLP (Time 0 sample; Figure 8A) as the control (i.e., for comparison of relative changes observed upon stress and the addition of APs).

For example, for the competitive ELISA results for AH-adsorbed HPV16 VLPs after exposure to stress conditions in the absence of APs (no-AP; Figure 8B), the HPV16 pentamer showed relatively intermediate structural perturbations (~34–66% mAb binding decrease relative to Time 0, colored light grey) for three of the known conformational epitopes (mAbs V5, A2, and 7E), while mAb binding to three surface linear epitopes (mAbs S1, J4, and H5) showed more severely disrupted sites (>66% binding loss, colored dark grey). Intriguingly, the binding of the E70 mAb, a conformational mAb with an unknown epitope, was substantially lower compared to the other conformational mAbs and mirrored the trends of the surface linear mAbs. These results indicate that certain regions on the HPV16 pentamer surface (i.e., epitopes associated with linear mAbs) were more susceptible to structural perturbations upon the exposure of the AH-adsorbed HPV16 VLP sample to stress conditions.

The competitive ELISA results for AH-adsorbed HPV16 VLPs under stress conditions in the presence of CB or MC are displayed in Figure 8C and Figure 8D, respectively. The combination of stress conditions and the addition of 27 mM CB (Figure 8C) yielded analogous results to the no-AP sample after stress exposure (Figure 8B). This result demonstrates that the addition of CB had minimal effects and did not synergistically enhance the structural perturbations observed under stress conditions. In contrast, for the same sample in the presence of 28 mM MC (Figure 8D), a markedly different mAb binding profile was observed in which the binding of the conformational mAbs V5, A2, and 7E and linear mAb H5 was substantially disrupted (>66% binding loss, colored dark grey), while the binding of the surface linear mAbs J4 and S1 was intermediately perturbed (34–66% mAb binding decrease, colored dark grey). The distinct mAb binding profile in the presence of MC, compared to CB and the no-AP samples, after storage of the AH-adsorbed HPV VLPs at an elevated temperature at pH 7.4 indicates that the destabilization mechanisms of the two APs are unique. Interestingly, the lack of binding to buried linear mAbs (B20 and D9) to any of the AH-adsorbed HPV16 VLP samples is consistent with regional but not global structural destabilization of the antigen under these conditions.

Similar comparisons were then made for the same unstressed and stressed AH-adsorbed HPV16 VLP samples in terms of the Cys residue accessibility results from the LC-MS peptide mapping assay. These results are also displayed schematically for the HPV16 pentamer in Figure 8. In comparison to the non-stressed control sample (Time 0; Figure 8A), for the stressed sample without AP (Figure 8B) or the stressed sample with CB (Figure 8C), Cys residue accessibility was only minimally affected (<33% loss). This result is distinct from the findings described above with the mAb binding studies, suggesting that the regional perturbations observed for mAb binding to the HPV16 VLP surface under these conditions were insufficient to obfuscate the cysteine residue accessibility to the small-molecular-weight alkylating agents. In contrast, the Cys accessibility was substantially perturbed following a 24 h at 50 °C incubation in the presence of MC (Figure 8D). For example, the size of regions observed to show changes in Cys residue accessibility disrupted in the presence of MC was larger and/or more dynamic compared to no-AP or CB stressed samples (colored in brown). The dramatic decrease in Cys accessibility in the MC sample warrants further investigation to better understand how the surface dynamics of the HPV16 VLP impact the accessibility of each individual Cys residue within the L1 proteins of the HPV16 pentamer.

Since CB and MC have similar log *p* values (2.03 and 1.96, respectively [14]), the larger destabilization induced by MC was likely driven by factors besides hydrophobicity, such as hydrogen bonding and π-π interactions [19,20,21]. The non-aromatic structure of CB could also be a factor in its minimally destabilizing effect on the HPV16 VLP. Future work could focus on investigating the amino acid residue composition of each binding epitope (and their surroundings sequences) to identify potential sites of interaction with APs and use this information to develop models to predict AP incompatibility with future vaccine antigens. Furthermore, the similarities and differences of the molecular mechanisms of CB and MC-induced destabilization of the other HPV VLP vaccine types (i.e., HPV6, HPV11, and HPV18) could also be examined.

### 4.5. Future Work Based on AP–Protein Interaction Studies with Recombinant Protein Therapeutics

In comparison to our results with AH-adsorbed HPV16 VLPs, mechanistic studies on the structural destabilization effects of APs used in multi-dose formulations of protein therapeutic candidates have been reported on a variety of smaller, globular proteins (in solution), including recombinant human growth hormone [21], recombinant human interleukin-1 receptor antagonist (rhIL-1ra) [20,44], recombinant human granulocyte colony stimulating factor (rhGCSF) [45], interferon alpha-2a (IFNA2), and cytochrome c [46,47,48]. In these studies, with APs other than TH, the interactions between APs and proteins were hypothesized to occur primarily through non-covalent hydrophobic forces [18,20,44,49]. Many of these APs (e.g., 2-phenoxyethanol, benzyl alcohol, phenol, methyl/propylparaben, and m-cresol) are phenolic or phenyl derivatives and are thus mostly hydrophobic in nature. Their aromatic groups can preferentially interact with hydrophobic patches on native and/or structurally altered proteins, thereby inducing conformational changes and/or increasing aggregation propensity. Interestingly, the addition of BA increased aggregation rates and caused minor disruptions in the tertiary structures of proteins but did not significantly alter the secondary structure and overall stability of proteins [20,44,50,51]. The relatively low binding affinities of APs to these proteins permit relatively straightforward mitigation strategies such as the addition of preferentially excluded excipients (i.e., sucrose), adjusting the pH and ionic strength of the buffer, or minimizing exposure to elevated temperatures [18].

The site-specific nature of the interactions of phenol (PH) or m-cresol (MC) with insulin has been extensively examined [52,53,54,55,56]. PH and MC are used as antimicrobial preservatives (APs) for commercial insulin drug products but interestingly can also act as stabilizers [56]. Phenols, and phenolic derivatives such as m-cresol and methyl parabens, have been shown to bind to a hydrophobic pocket on the monomer–monomer interface within the hexameric form of insulin [55,56]. Overall, the interaction between these APs and insulin is hydrophobic in nature and driven by enthalpy [52]; however, other forces such as van der Waals forces, ring stacking, and H-bonding are involved [53,54,55,57]. DSC studies have demonstrated that both phenol and m-cresol can bind to three sites on the insulin hexamer [55]. In addition, the -OH group of phenol can form H-bonds to the carbonyl oxygen and amide nitrogen on the nearby cysteines in the A6 and A11 regions of insulin [54,57]. Studies on crystallized insulin show that the aromatic ring in phenol can also form ring stacking structures with a histidine residue on the B5 chain [54]. MC binding behaves similarly to PH, albeit with some structural differences due to the additional methyl group. For example, studies on crystallized insulin in the presence of MC indicate van der Waals interactions between the aromatic ring of MC and the aspartic acid residue at B28; moreover, the methyl group of MC also shows hydrophobic interactions with three amino acid residues (tyrosine at B26, isoleucine at A2, and valine at A3) [54]. These weak, non-covalent interactions between insulin and phenol/m-cresol all cumulatively structurally stabilize the hexamer form of insulin and protect it from degradation during storage.

Given this extensive knowledge of AP interactions with insulin, future work in computational modelling and high-resolution structural techniques (e.g., NMR, X-ray crystallography, hydrogen–deuterium exchange mass spectrometry) could potentially identify the precise molecular interactions/forces between APs and HPV VLPs. Future applications of these two analytical techniques could include evaluating other HPV serotypes which have different susceptibility to APs, with some (HPV6 and 11) being more resistant while others (HPV18) are more vulnerable to AP-induced destabilization. Mapping unknown binding epitopes for serotype-specific conformational mAbs (e.g., E70 for HPV16) would also contribute to a greater coverage of the VLP surface to better understand how APs destabilize HPV VLPs. Additional future studies could also investigate other analytical approaches including binding enthalpies of APs to HPV VLPs via isothermal titration calorimetry and higher-resolution techniques (listed above) to capture more subtle dynamic changes on the VLP surface in the presence of APs. To this end, the use of molecular simulations could potentially also offer insights into specific sites/patches of interaction between the VLP surface and various APs. The findings from such mechanistic studies of AP interactions with antigens would not only help refine HPV vaccine multi-dose formulation strategies but also optimize analytical characterization tools to facilitate future work with other recombinant protein vaccine antigens to be formulated in the presence of adjuvants and APs.

## 5. Conclusions

In this work, we investigated two complementary analytical approaches to better understand the impact of two antimicrobial preservatives (APs), namely chlorobutanol (CB) or m-cresol (MC), on the structural integrity and stability of the Alhydrogel™ (AH)-adsorbed HPV16 VLP antigen. First, we performed competitive ELISA assays with a panel of nine different mAbs (that bind different conformational, surface linear, and buried linear epitopes on HPV16 VLPs) to provide greater insight into structural alterations within the AH-adsorbed HPV16 VLPs induced by stress conditions (elevated temperature at pH 7.4) in the presence and absence of CB and MC. Second, we developed an LC-MS peptide mapping assay coupled with differential alkylation to quantify the structural alterations in the same AH-adsorbed HPV16 VLP samples by monitoring the accessibility/redox state of the Cys residues in each L1 protein within in the HPV16 VLPs. Since there are 12 Cys residues located throughout the L1 protein monomer, and there are 72 pentamers of the L1 protein in an HPV16 VLP, this assay tracks at a total of 4320 Cys residues in the HPV16 VLP antigen. Moreover, this analysis works for samples where the antigen is adsorbed to an aluminum-salt adjuvant. The combination of results from these two analytical approaches demonstrated more notable structural alterations induced within the HPV16 VLPs (adsorbed to AH adjuvant) upon exposure to elevated temperature in the presence of MC (vs. no-AP and CB), results that correlated well with trends in the accelerated storage stability data.

The combined results from the immunoassays (using a panel of mAbs against various epitopes) and the LC-MS peptide mapping analyses (for Cys residue accessibility/redox) provided a “regional-level” perspective on the structural integrity of aluminum-adjuvanted protein antigens upon exposure to stresses such as elevated temperature and the addition of APs. These two methods bridge the gap between commonly used, lower-resolution biophysical techniques that monitor “global” structural alterations in antigens (e.g., DSC, fluorescence spectroscopy, FTIR) and more recent work with highly specialized, higher-resolution analytical approaches that monitor “local” structural changes (e.g., cryo-electron microscopy [13], nuclear magnetic resonance spectroscopy [58]). To this end, this work demonstrates the utility of “regional-level” structural analysis methods, as part of vaccine multi-dose formulation development, to provide a better understanding of structural alterations caused by APs occurring within complex vaccine protein antigens (adsorbed to aluminum-salt adjuvants) using more easily accessible analytical techniques.

## Data Availability

All data associated with this study are available from the corresponding author(s). The datasets generated and/or analyzed are also available in the KU ScholarWorks repository, https://doi.org/10.17161/1808.35060.

## Supplementary Materials

The following supporting information can be downloaded at: https://www.mdpi.com/article/10.3390/vaccines12060580/s1, Figure S1: Stability profile of AH-adsorbed, quadrivalent HPV VLP serotypes (HPV6, HPV11, HPV16, and HPV18) at different pH values after incubation at 50 °C as measured by competitive ELISA; Figure S2: Representative competitive ELISA binding curves of the D9 mAb on HPV16 VLP antigen formulated with AH-adjuvant (adsorbed) in the absence or presence of APs Time 0, Table S1: Summary of the optimization of method parameters for each of the AH-adsorbed HPV16 VLP Competitive ELISA assays developed for each of the conformational and linear HPV16 mAbs.

## Abbreviations

2-PE	2-phenoxyethanol
AH	Alhydrogel^TM^
AP	Antimicrobial preservative
BA	Benzyl alcohol
CB	Chlorobutanol
DSC	Differential scanning calorimetry
ELISA	Enzyme-linked immunosorbent assay
EM	Electron microscopy
HPV	Human papillomavirus
IAM	Iodoacetamide
LC-MS	Liquid chromatography–mass spectrometry
LMICs	Low- and middle-income countries
MC	m-cresol
MP	Methyl paraben
NEM	N-ethylmaleimide
PH	Phenol
PP	Propyl paraben
PTM	Post-translational modification
T0	Time zero
TH	Thimerosal
VLP	Virus-like particle
