# Supplementary material for: A Combined LC-MS and Immunoassay Approach to Characterize Preservative-Induced Destabilization of Human Papillomavirus Virus-like Particles Adsorbed to an Aluminum-Salt Adjuvant
Source: Vaccines (Basel). 2024 May 26;12(6):580. doi: 10.3390/vaccines12060580 (PMC11209183; doi:10.3390/vaccines12060580)
Supplement: Supplementary file 1 [file vaccines-12-00580-s001.zip › vaccines-2953856-supplementary.pdf]

SUPPLEMENTAL INFORMATION

A Combined LC-MS and Immunoassay Approach to Characterize Preservative-Induced Destabilization of Human Papillomavirus Virus-like Particles Adsorbed to an Aluminum-Salt Adjuvant

Supplemental Materials and Methods

Materials- HPV16 VLPs in bulk solution were produced by Serum Institute of India, Pvt. Ltd. and kept at –80°C until use. Conformational and linear mAbs for HPV16 (H16.V5, H263.A2, H16.7E, H16.E70, H16.J4, H16.S1, H16.H5, H16.B20, and H16.D9) were produced by the laboratory of Neil Christensen (Penn State University) as described previously [1,2]. Aluminum hydroxide adjuvant (Alhydrogel®, AH) was purchased from InvivoGen (San Diego, CA, USA). Sodium chloride was purchased from Fisher Scientific (Waltham, MA, USA). Polysorbate 80 was purchased from Thermo Scientific (Rockford, IL, USA). L-Histidine, 2-phenoxyethanol, phenol, m-cresol, benzyl alcohol, and thimerosal were purchased from Sigma Aldrich (St. Louis, MO, USA). Propylparaben, methylparaben, and chlorobutanol were purchased from Spectrum Chemicals (Gardena, CA, USA).

HPV VLP adsorption to AH- Bulk HPV VLPs were adsorbed to AH by incubating the mixture for 24-48 hrs. at 4°C and then buffer exchanged four times into a formulation buffer (20 mM Histidine, 250 mM NaCl, 0.025% polysorbate 80, pH 7.4). The exchange consisted of using gentle centrifugation (100 x g for 15 mins) and manual removal of ~65% of the resulting supernatant, followed by replacement of an equal volume of fresh formulation buffer. The formulated and adjuvanted samples consisted of 0.05 mg/mL each HPV VLP serotype (6, 11, 16, 18), for a final concentration of 0.2 mg/mL HPV VLPs adsorbed to 0.72 mg/mL aluminum (from AH) in the formulation buffer (see above). APs were added from concentrated stock solutions such that the final concentrations were either 27 mM CB; 28 mM MC; 53 mM PH; 72 mM 2-PE; 93 mM BA; 11 mM MP + 1 mM PP; or 0.25 mM TH.

Accelerated stability studies- An initial stability evaluation was carried out in samples of quadrivalent AH-adsorbed HPV VLP sample (HPV L1 serotypes 6, 11, 16, and 18) in formulation buffer (see above) at pH 6.5, 7.0, or 7.4, and then incubated for 5 weeks at 4 or 50°C. Samples were prepared in duplicate; for each sample, 3 mL was filled into sterile 5 mL Eppendorf tubes which were capped and sealed with parafilm. Aliquots were removed for each time point. *In vitro* potency was measured for each serotype using a competitive ELISA assay (described below). For stability studies with monovalent HPV16 VLPs, 0.2 mg/mL antigen was adsorbed to AH formulation buffer (pH 7.4) as described above; either CB (final concentration: 27 mM) or MC (final concentration: 28 mM) was added, then incubated for 24 hrs. at 4 or 50°C. The AH-adjuvanted HPV16 VLPs samples were prepared in duplicate; for each sample, 350 µL was filled into sterile 1.5 mL Eppendorf tubes and sealed with Parafilm.

Differential scanning calorimetry (DSC)- DSC analysis of HPV VLPs has been previously described in detail [3]. Briefly, a MicroCal Auto-VP DSC II (Malvern Panalytical, Malvern, UK) was used to analyze AH-adsorbed HPV VLP samples (0.4 mg/mL HPV16, 0.72 mg/mL aluminum (from AH) in formulation buffer, pH 7.4) from 10-90°C with a scan rate of 60°C/hr. All parameters were similar to the referenced method except that the concentration of HPV16 VLP was increased to 0.4 mg/mL in this study to achieve better signal-to-noise ratio. Samples were analyzed in duplicate for each run, with a total of three runs. Buffer subtraction and concentration normalization were performed using Origin2020.

Competitive ELISA- A competitive ELISA method to assess binding to a subset of type-specific monoclonal antibodies to AH-adjuvanted HPV VLPs has been described in detail elsewhere [3].

Briefly, HPV VLPs in bulk solution (i.e., not adsorbed to Alhydrogel) were diluted 1:500 in DPBS and coated onto 96-well ELISA plates overnight (50 µL in each well). The following day, test samples and reference standards were diluted in blocking buffer (1% casein in TBS + 0.05% PS-20) for an hr. at 4°C. The quadrivalent AH-adsorbed HPV VLP drug product was assayed using type-specific neutralizing mAbs for each serotype: M48 for HPV6, H3 for HPV11, V5 for HPV16, and J4 for HPV18. The working concentrations of these mAbs have been reported previously [4,5]. The rest of the assay proceeds as described elsewhere (supplementary materials) [3].

In addition to the assay described above for each of the four HPV serotypes, in this work, we developed similarly formatted competitive ELISA assays using additional conformational (H263.A2, H16.E70, and H16.7E), surface linear (H16.J4, H16.H5, and H16.S1), and buried linear (H16.D9 and H16.B20) mAbs for monovalent AH-adsorbed HPV16 VLP drug product. For the buried linear mAbs (H16.D9 and H16.B20), a disassembled HPV16 VLP was used as the reference standard: AH-adsorbed HPV16 VLPs were mixed with an equal volume of denaturing/disassembly buffer (0.5 M carbonate, 1 mM DTT, pH 10) and incubated at 37°C overnight. The following day, samples and reference standards were diluted in blocking buffer (1% casein in TBS + 0.05% PS-20) for 1 hr. at 4°C. The target HPV16 protein concentration and the mAb working concentration depended on the mAb being tested (see **Supplementary Table S1**). The rest of the assay proceeded as described above and in detail elsewhere [3]. Data processing was performed using Origin 2020 by using a four-parameter logistic fit (4 PL) equation (using a no weighting fit) [3].

SDS-PAGE- SDS-PAGE analysis of HPV VLPs has been previously described in detail [3]. Briefly, approximately each AH-adsorbed HPV16 VLP sample (analyzed in duplicate) was treated with 0.4 M sodium phosphate pH 8.0 and 50 mM DTT, and then incubated at 98°C for 5 min in

the presence of lithium dodecyl sulfate buffer. Approximately 240 ng of each HPV16 L1 sample was analyzed using a 4-12% Bis-Tris gel (Invitrogen), visualized using Pierce™ Silver Stain Kit (Thermo Scientific), and analyzed using AlphaView Software (ProteinSimple).

Cysteine accessibility assay using differential alkylation followed by enzymatic digestion- Approximately 20 µg of each AH-adsorbed HPV16 VLP sample were analyzed in triplicate. The samples (0.2 mg/mL HPV16 L1, 0.72 mg/mL AH in formulation buffer, pH 7.4) were incubated for 1 hr. at 37°C with the first alkylating agent, 10 mM NEM. NEM-alkylation, and then quenched by adding 70 mM L-Cysteine (Sigma-Aldrich) and incubating at room temperature for 10 min. The samples were then centrifuged for 1 min at 4,000 x g, the supernatant was discarded, and the AH-adsorbed HPV16 VLP pellet was washed twice with 0.5M Bis-Tris Propane pH 7.4. HPV16 L1 protein was then desorbed from the AH by adding 5% SDS, 133 mM sodium phosphate pH 8.0, and 3 mM TCEP, and incubating for 10 min at 98°C. The samples were centrifuged for 2 min at 10,000 x g and the resulting supernatants were transferred to fresh Eppendorf tubes containing a second alkylating agent, 15 mM IAM (Thermo Scientific), to modify the remaining Cys residues not already alkylated with NEM. The HPV16 L1 proteins were then denatured, isolated, and trypsin-digested using a commercial kit and associated protocol (S-Trap™ micro kit, Protifi LLC). The resulting HPV16 L1 peptides were subjected to identification and quantification using LC-MS peptide mapping as described below.

LC-MS Peptide Mapping- Approximately 10 µg of HPV16 L1 peptides were injected onto an Acquity Premier column (2.1 x 150 mm, 1.7 µm, Waters Corporation) maintained at 60°C within a 1290 Infinity II UHPLC system (Agilent Technologies). Peptides were eluted from the column

using a 65 min 0-45%B gradient (mobile phase A: 0.1% formic acid in water, mobile phase B: 0.1% formic acid in acetonitrile) at a flow rate of 0.2 mL/min and then subjected to MS analysis using a 6545XT QTOF mass spectrometer (Agilent Technologies). Electrospray ionization parameters consisted of: 325°C gas temperature, 4,000V Vcap, and 100V fragmentor. Mass spectra were collected from 250-1700 m/z at 1 spectra/sec. The threshold for MS/MS analysis was 10,000 counts and the two most abundant ions were selected for CID fragmentation per cycle. Mass spectra were processed using MassHunter Bioconfirm v10.0 software (Agilent Technologies), with the following variable modifications included: Cys NEM alkylation, Cys IAM alkylation, Met oxidation, Asn deamidation, N-terminal acetylation, and Ser/Thr/Tyr phosphorylation.

Data visualization and Statistical Analysis- The presented figures and cartoon models were generated using GraphPad Prism 9.0.0 (GraphPad Software, LLC), Origin 2020 (OriginLab, Northampton, MA, USA), ProteinImager (https://3dproteinimaging.com), BioRender (https://www.biorender.com/), and PyMol (Warren Lyford DeLano/Schrödinger, LLC). Statistical analysis was performed using a Student's t-test with a p-value of <0.05 considered as significant.

Supplemental References

1. Christensen, N.D.; Dillner, J.; Eklund, C.; Carter, J.J.; Wipf, G.C.; Reed, C.A.; Cladel, N.M.; Galloway, D.A. Surface conformational and linear epitopes on HPV-16 and HPV-18 L1 virus-like particles as defined by monoclonal antibodies. *Virology* **1996**, *223*, 174-184, doi:10.1006/viro.1996.0466.
2. Christensen, N.D.; Reed, C.A.; Cladel, N.M.; Hall, K.; Leiserowitz, G.S. Monoclonal antibodies to HPV-6 L1 virus-like particles identify conformational and linear neutralizing epitopes on HPV-11 in addition to type-specific epitopes on HPV-6. *Virology* **1996**, *224*, 477-486, doi:10.1006/viro.1996.0554.
3. Jerajani, K.; Wan, Y.; Hickey, J.M.; Kumru, O.S.; Sharma, N.; Pullagurla, S.R.; Ogun, O.; Mapari, S.; Whitaker, N.; Brendle, S.; et al. Analytical and Preformulation Characterization Studies of Human Papillomavirus Virus-Like Particles to Enable Quadrivalent Multi-Dose Vaccine Formulation Development. *J Pharm Sci* **2022**, *111*, 2983-2997, doi:10.1016/j.xphs.2022.07.019.
4. Jerajani, K.; Wan, Y.; Kumru, O.S.; Pullagurla, S.R.; Kumar, P.; Sharma, N.; Ogun, O.; Mapari, S.; Brendle, S.; Christensen, N.D.; et al. Multi-Dose Formulation Development for a Quadrivalent Human Papillomavirus Virus-Like Particle-Based Vaccine: Part I - Screening of Preservative Combinations. *J Pharm Sci* **2023**, *112*, 446-457, doi:10.1016/j.xphs.2022.09.001.
5. Sharma, N.; Jerajani, K.; Wan, Y.; Kumru, O.S.; Pullagurla, S.R.; Ogun, O.; Mapari, S.; Brendle, S.; Christensen, N.D.; Batwal, S.; et al. Multi-dose Formulation Development for a Quadrivalent Human Papillomavirus Virus-Like Particle-Based Vaccine: Part II-Real-time and Accelerated Stability Studies. *J Pharm Sci* **2023**, *112*, 458-470, doi:10.1016/j.xphs.2022.11.021.

Supplementary Table S1. Summary of the optimization of method parameters for each of the AH-adsorbed HPV16 VLP Competitive ELISA assays developed for each of the conformational and linear HPV16 mAbs. See Supplemental methods section for details of the Competitive ELISA format and composition of the blocking buffer.

<table>
<tr><th>HPV16 primary mAb</th><th>HPV16 primary mAb working conc. (µg/mL)</th><th>Target conc. of HPV16 VLP reference std/sample in blocking buffer (µg/mL)</th><th>Starting conc. of blocked HPV16 VLP sample in initial row of assay plate prior to serial dilution (µg/mL)</th></tr>
<tr><td>H16.V5</td><td>10</td><td rowspan="6">17</td><td rowspan="5">6</td></tr>
<tr><td>H16.E70</td><td>10</td></tr>
<tr><td>H16.7E</td><td>20</td></tr>
<tr><td>H263.A2</td><td>20</td></tr>
<tr><td>H16.J4</td><td>10</td></tr>
<tr><td>H16.H5</td><td>20</td><td>11</td></tr>
<tr><td>H16.S1</td><td>20</td><td rowspan="3">100</td><td>80</td></tr>
<tr><td>H16.D9</td><td>20</td><td rowspan="2">30</td></tr>
<tr><td>H16.B20</td><td>10</td></tr>
</table>

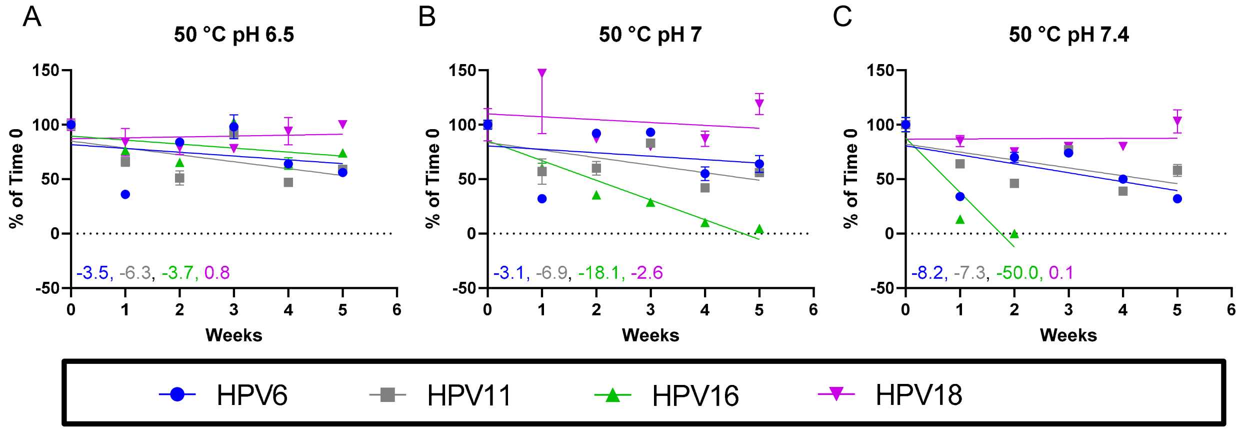

Supplemental Figure S1. Stability profile of AH-adsorbed, quadrivalent HPV VLP serotypes (HPV6, HPV11, HPV16, and HPV18) at different pH values after incubation at 50°C as measured by competitive ELISA. (A) pH 6.5, (B) pH 7.0, or (C) pH 7.4. The error bars represent the range from two replicates and the slopes of linear regression fits are reported. The change in slope per week is shown at the bottom for each HPV serotype (blue = HPV6, grey = HPV11, green = HPV16, and pink = HPV18). Description of formulation composition and stability study conditions are provided in the Supplemental methods section. Type-specific mAbs used for each serotype are as follows: H6.M48 for HPV6; H11.H3 for HPV11; H16.V5 for HPV16; and H18.J4 for HPV18.

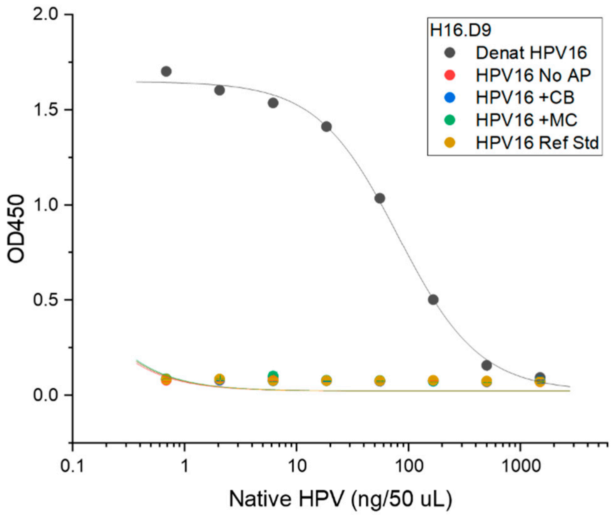

Supplemental Figure S2. Representative competitive ELISA binding curves of the D9 mAb on HPV16 VLP antigen formulated with AH-adjuvant (adsorbed) in the absence or presence of APs Time 0. Note that the extensively denatured HPV16 VLP sample (positive control) displayed a sigmoidal curve, while all the other samples, including the reference sample did not bind to the D9 mAb.
